# Social isolation of aged mice drives dramatic release of inflammatory lipoxygenase-derived oxylipins

**DOI:** 10.1038/s41514-026-00405-6

**Published:** 2026-05-16

**Authors:** Mareike Wichmann-Costaganna, Raphaëlle Petit, Julia Lindner, Madlen Haase, Vivien Bachmann, Robert Klaus Hofstetter, Markus Werner, Christiane Frahm, Oliver Werz, Patrick Schädel

**Affiliations:** 1 Department of Pharmaceutical/Medicinal Chemistry, Institute of Pharmacy, Friedrich Schiller University Jena, Jena, Germany; 2 Department of Neurology, Jena University Hospital, Jena, Germany; 3 Department of Hematology, Cellular Therapy, Hemostaseology and Infectious Diseases, University Hospital Leipzig, University of Leipzig Medical Center and Comprehensive Cancer Center Central Germany (CCCG) Leipzig-Jena, Leipzig, Germany

**Keywords:** Diseases, Immunology, Physiology

## Abstract

Oxylipins, signalling molecules derived from polyunsaturated fatty acids, act as key mediators controlling inflammatory processes. Ageing fuels the disruption of this network, promoting inflammageing. Social isolation, a common feature of ageing, may contribute to the emergence of pro-inflammatory responses, further aggravating conditions like cognitive decline and frailty. Here, we studied how repeated social isolation impacts inflammation-related oxylipin profiles in seven different organs and serum of aged mice. Additionally, we explored physical exercise as a tool to ameliorate age- and isolation-associated inflammation. Our results show that social isolation induces significant increases in interleukin-1β levels and stimulates a dramatic production of lipoxygenase (LOX)-derived oxylipins in an organ-dependent manner, particularly pronounced in liver, lung, and spleen. Physical exercise failed to mitigate the pro-inflammatory effects induced by social isolation. These effects did not occur in the circulatory system as serum oxylipin levels remained relatively unchanged by isolation. The unexpected and striking elevation of oxylipins across the organs highlights the detrimental effect of social isolation and proposes key roles of oxylipins in stress-related inflammageing.

## Introduction

Ageing is a complex, multifactorial biological and social process, accompanied by the accumulation of deleterious changes often resulting in decreased health, cognitive decline, and overall loss of function and fitness^1,2^.

Disruption of homeostasis, caused by impaired immune functions, immunosenescence^3^, and subsequent increase in chronic low-grade inflammation, termed inflammageing^4,5^, is considered one of the proposed hallmarks of ageing^6^. Inflammatory processes are tightly regulated by a network of mediators, such as cytokines, chemokines, and oxylipins^7^. During ageing, this complex network becomes dysregulated and aggravates inflammatory processes, as oxylipins play vital roles as lipid mediators in facilitating the initiation, perpetuation, and resolution of inflammation^8,9^. Oxylipins are produced from polyunsaturated fatty acids (PUFA) including omega-6 (e.g., arachidonic acid (AA, C20:4)) and omega-3 (e.g., docosahexaenoic acid (DHA, C22:6), eicosapentaenoic acid (EPA, C20:5) members. Their mode of action can be both pro- and anti-inflammatory: while pro-inflammatory AA-derived prostaglandins (PG) and leukotrienes (LT) are biosynthesised via the cyclooxygenase (COX) and 5-lipoxygenase (LOX) pathway, anti-inflammatory and pro-resolving oxylipins are mainly omega-3-PUFA-derived and are generated by 5-, 12-, and 15-LOX. The latter oxylipins encompass specialized pro-resolving mediators (SPM) such as protectins, lipoxins (LX), maresins (MaR), resolvins (Rv), as well as their mono-hydroxylated precursors (e.g., 15-hydroxy-eicosapentaenoic acid (15-HEPE), 17-hydroxy-docosahexaenoic acid (17-HDHA))^10,11^. Aberrant oxylipin biosynthesis is a characteristic feature of several age-related diseases, such as cardiovascular disease^12,13^, atherosclerosis^14^, and neurodegeneration^15,16^. In recent studies, we revealed that oxylipin formation exhibits unique organ- and cell type-specific signatures^17,18^, underlining the necessity to consider age as variable when investigating inflammation.

Furthermore, lifestyle crucially determines biological health and the severity of ageing^19,20^. Social isolation and loneliness are directly associated with poor health outcomes, increased inflammation, and cognitive decline in ageing^21,22^. A meta-analysis of 148 studies on social relationships and mortality risks has found that individuals with stronger social relationships exhibit a higher likelihood of survival^23^. Moreover, genome-wide analyses revealed that people experiencing a high degree of subjective social isolation show increased leukocyte transcriptional activity, especially in pro-inflammatory pathways, directly linking social isolation with inflammation^24^. Maintaining social connections in old age has proven to be particularly beneficial, as the negative impact of social isolation is estimated to be higher than that of diabetes^25^. Mouse studies on social isolation show elevated corticosterone levels, which acts as the main active endogenous glucocorticoid in mice, indicating a physiological stress response, along with increased inflammation and impaired cognitive and immune function^26–28^. Conversely, a positive social environment is associated with better immunity and increased lifespan^29^, underscoring the critical role of social connections in both ageing and inflammatory processes. But how social isolation affects oxylipin networks and which roles oxylipins play in physiological consequences of social isolation is unknown.

Metabolic interventions such as voluntary exercise (e.g., for humans taking a walk, for mice voluntary wheel running) are well established as a tool that may prolong the life and health span of an individual^30^ and subsequently may mitigate the negative impact of other lifestyle factors like isolation. While regular exercise yields a multitude of positive outcomes, the exact molecular drivers behind these benefits are not yet fully understood. Exercise not only decreases the secretion of pro-inflammatory cytokines but also shifts the oxylipin pathways toward omega-3 substrates, leading to elevated levels of SPM and their precursors^31,32^. Additionally, it was found that exercise reduces perceived loneliness^33^. Although previous studies have examined inflammageing, age-related social isolation, and late-life exercise interventions individually, the combined effects and interactions of these factors remain unexplored. Here, we elucidated the implications of age-associated social isolation, defined as absence or reduction of social contact, and related stress on the inflammatory microenvironment in aged mice, focusing on oxylipins, as well as cognitive function, and whether accompanied exercise (i.e., voluntary wheel running) can mitigate the detrimental effects of social isolation. We accounted for differential plasticity in various organs by screening seven different organ/tissue types, as well as serum (systemic circulation), creating a fundamental framework linking inflammation, ageing, and age-associated isolation stress.

## Results

**Fig. 1:**
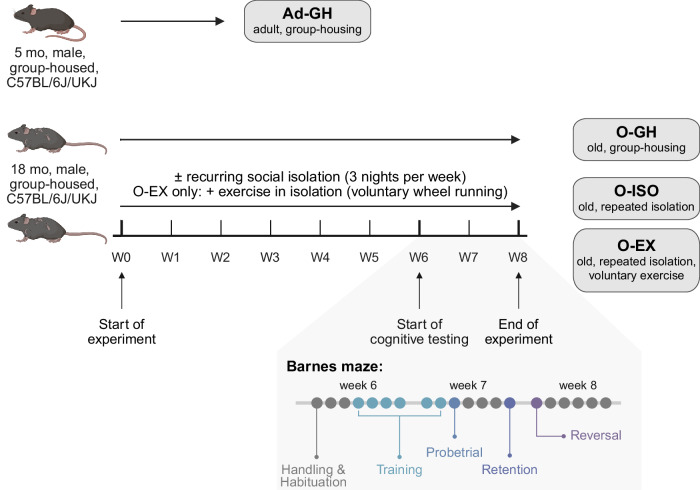
Schematic representation of the recurring social isolation and physical exercise intervention. 5-month-old, adult male mice (Ad-GH, *n* = 5) and 18-month-old, aged male mice (O-GH, *n* = 6) were kept in group housing until sacrificed. To investigate the effect of recurring social isolation in late-life, 18-month-old mice were repeatedly isolated into single cages for 3 individual nights per week, and resocialised with their corresponding littermates throughout a total of 8 subsequent weeks. The single cages were equipped with (=O-EX) or without (=O-ISO) running wheels. Aged mice underwent cognitive testing (*n* = 17–18) during the last weeks before the end of the experiment. Organs and blood for serum investigation (*n* = 5–6) were collected immediately after sacrifice. Scheme was created in BioRender (Schädel, P. (2026) https://BioRender.com/o33h2ks).

The inflammatory status of 7 organs (brain, heart, fat, liver, lung, muscle, spleen) and serum of adult (5 months) and aged (20 months) C57BL6/J/UKJ mice was assessed by analysis of inflammation-related cytokine and oxylipin profiles. To investigate the effect of social isolation stress at a later stage in life, we recurrently separated mice at an age of 18 months into single cages for three individual nights per week over a period of 8 weeks. Moreover, we addressed exercise as an established, anti-inflammatory metabolic intervention by providing a running wheel to a subgroup of isolated aged mice. In this context, aged mice additionally underwent a Barnes maze test to assess intervention-related changes in cognition. The detailed experimental setup is depicted in Fig. 1.

### Organ-specific inflammatory cytokines are differentially affected by ageing but significantly induced by recurring social isolation

**Fig. 2:**
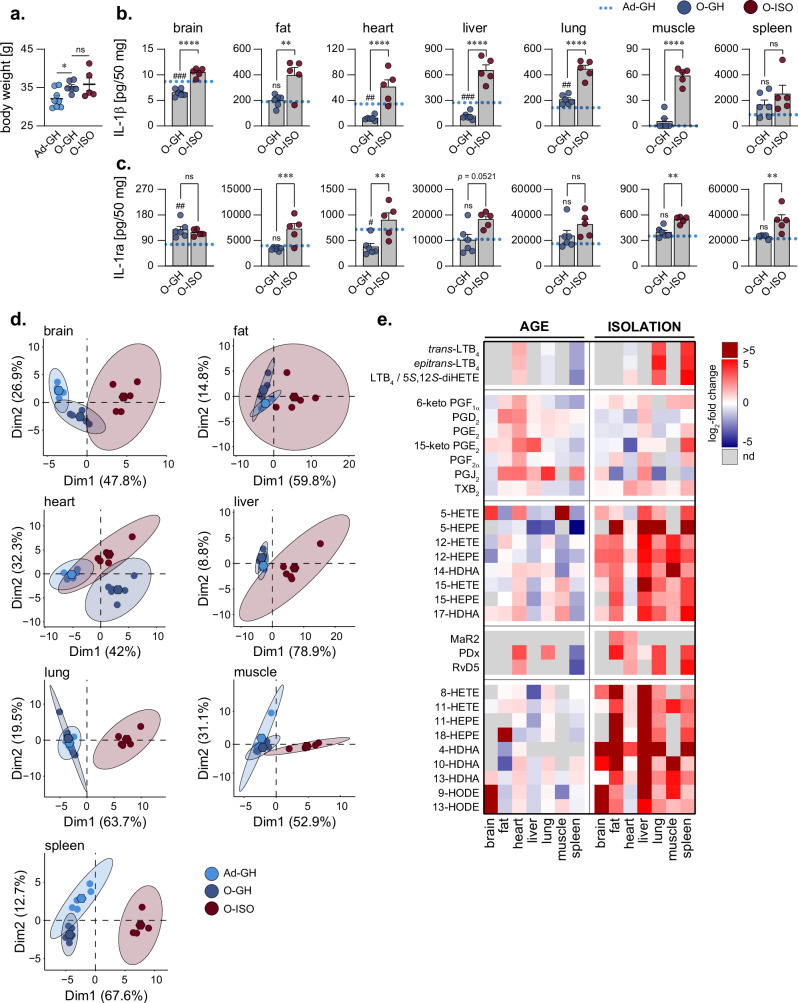
Ageing leads to organ-specific inflammatory phenotypes that are aggravated by social isolation stress. **a** Body weight of adult (5 months) and aged (20 months) mice after being kept in group-housing (Ad-GH and O-GH, respectively) or repeatedly isolated (O-ISO). **b**, **c** Concentration of the cytokines IL-1β and IL-1ra in pg per 50 mg organ. The blue dotted line represents mean levels in adult, group-housed (Ad-GH) mice. Values that could not be computed were set to 0. **d** Principal component analysis of organ-specific oxylipin profiles. Hexagons indicate the mean PCA score of all screened replicates within respective experimental groups. **e** Log_2_-fold changes for the organ-specific concentrations of individual oxylipins for the comparison of aged (O-GH) and adult (Ad-GH) mice kept in group-housing (left panel), and the comparison of aged, repeatedly isolated (O-ISO) and aged, group-housed (O-GH) mice (right panel). Fold-changes that could not be calculated due to missing values (e.g., below limit of detection = nd) are depicted in grey. Statistics: Data are shown as (**a**–**c**) mean ± SEM. The number of biological replicates is *n* = 5 for Ad-GH and O-ISO, *n* = 6 for O-GH. One-way ANOVA with post-hoc Šídák’s multiple comparisons test with or without Brown-Forsythe and Welch correction was performed for the indicated comparisons, with # for the comparison of O-GH versus Ad-GH and * for the comparison of O-ISO versus O-GH.

Ageing significantly increased (*p* = 0.018) the body weight of the mice. Old mice in group-housing (O-GH) had a mean weight of 35.2 ± 0.6 g, while the mean weight of adult mice in group-housing (Ad-GH) was 32.1 ± 0.9 g. Social isolation stress in old mice due to repeated isolation into single cages (O-ISO) did not significantly alter the final body weight (35.9 ± 1.7 g, *p* = 0.692). However, the body weight of these isolated aged mice (O-ISO) showed a higher degree of variation versus old mice in group-housing (O-GH) (Fig. 2a).

To address the inflammatory status of the investigated organs, we examined the protein level of the pro-inflammatory cytokine interleukin (IL)-1β, that acts as a robust marker of a chronic inflammatory microenvironment, IL-6, IL-12 p70, tumour necrosis factor alpha (TNF-α) and the IL-1β counteractor IL-1 receptor antagonist (IL-1ra). We found that ageing did not markedly change IL-1β levels in fat, muscle, and spleen (Fig. 2b). However, IL-1β levels were significantly decreased due to ageing in brain (Ad-GH: 8.7 ± 0.2, O-GH: 6.5 ± 0.3 pg/50 mg), heart (Ad-GH: 34.5 ± 5.3, O-GH: 12.3 ± 1.5 pg/50 mg), and liver (Ad-GH: 273.7 ± 31.7, O-GH: 117.5 ± 17.6 pg/50 mg). Only in the lung, IL-1β was significantly elevated (Ad-GH: 143.5 ± 8.1, O-GH: 205.9 ± 14.6 pg/50 mg) in the aged compared to the adult mice (Fig. 2b). Similarly, the levels of the additional pro-inflammatory cytokines IL-6, IL-12 p70 and TNF-α remained largely unaltered or significantly decreased due to ageing, only muscle displayed significantly increased levels of IL-12 p70 following ageing compared to adult mice (Supplementary Fig. S1). When the aged mice underwent social isolation (O-ISO), however, IL-1β significantly increased in all studied organs except in spleen with only minor elevation versus the O-GH mice (Fig. 2b, 1.8-fold, *p* = 0.309). Again, other pro-inflammatory cytokines revealed comparable dynamics following isolation with strongly increased levels observed in fat, heart, liver and muscle homogenates (Supplementary Fig. S1). Overall, this translates to a distinct, organ-specific ageing-related impact on the secretion of pro-inflammatory cytokines, while stress due to isolation seems to consistently promote inflammation in most organs. A similar pattern was observed for the levels of IL-1ra, being significantly increased following isolation of aged mice in fat, heart, liver, muscle, and spleen (Fig. 2c).

### Social isolation potently induces lipoxygenase-derived oxylipin formation

Since oxylipins are crucial regulators of all stages of inflammation^10,34,35^, we assessed the oxylipin signature profiles of the different organs using a targeted lipidomic approach based on ultra-performance liquid chromatography coupled to tandem mass spectrometry (UPLC-MS/MS). To get an overview of age- and isolation-related changes in the complex oxylipin profiles, we performed an unbiased principal component analysis. This analysis revealed distinct age-related alterations in the oxylipin profiles for brain, fat, heart, and spleen between both group-housed adult (Ad-GH) and aged mice (O-GH) (Fig. 2d). An even more pronounced separation of the clusters for all organs was observed following isolation of aged mice (O-ISO) and only in the heart, age had a stronger effect on oxylipin profiles than isolation (Fig. 2d).

Looking at the changes of the individual screened oxylipins due to ageing, we found distinct organ-specific ageing signatures with liver, muscle, and spleen exhibiting an overall decrease in oxylipins, whereas brain, fat, and lung showed divergent oxylipin profiles with age (Fig. 2e, left). In the heart, ageing led to increased amounts of oxylipins, particularly pronounced for PGs (Fig. 2e, left). Social stress in aged mice due to isolation, however, drives a remarkable increase of a broad spectrum of oxylipins, especially derived from 5-, 12-, and 15-LOX across various organ systems (Fig. 2e, right). Interestingly, in lung and spleen, the inflammation-resolving SPMs RvD5 and PDX produced by 12/15-LOX were elevated along with pro-inflammatory LTs derived from 5-LOX. In the brain and heart, the oxylipin levels were mostly unaffected by isolation of the aged mice (Fig. 2e, right).

**Fig. 3:**
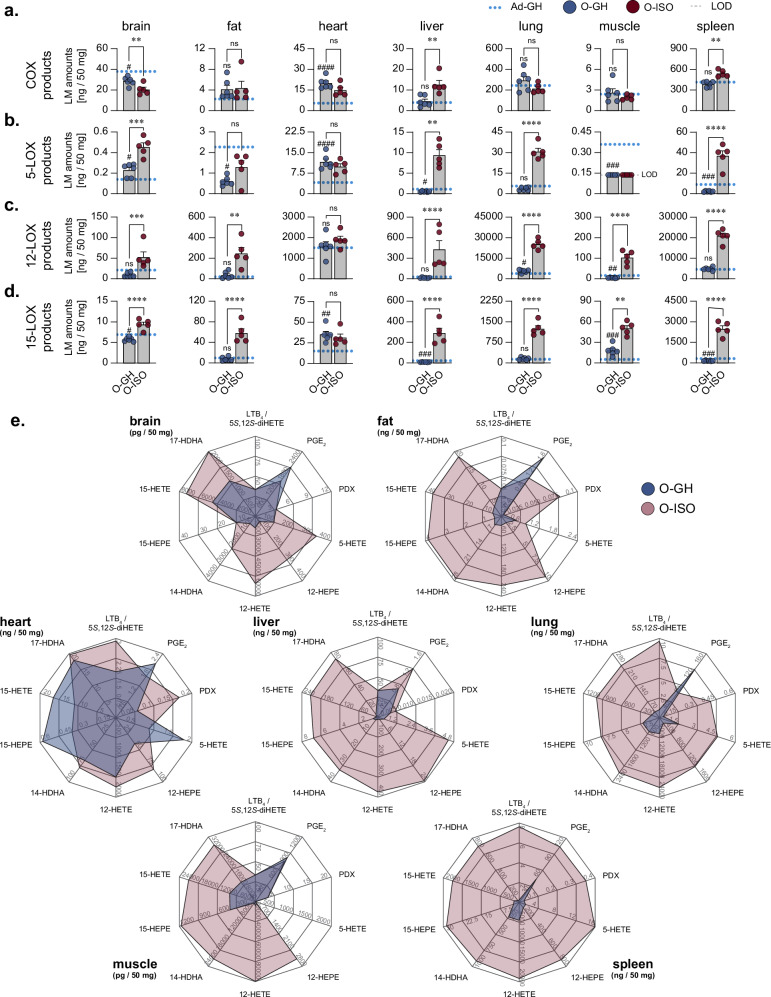
Social isolation stress leads to induction of LOX pathways. Total grouped amounts of oxylipins that are products of the COX (**a**) or LOX (**b**–**d**) pathways. Metabolites were grouped as follows: *COX* – PGD_1_, PGE_1_, PGF_1α_, 6-keto PGF_1α_, PGD_2_, PGE_2_, 15-keto PGE_2_, PGF_2α_, PGF_2ß_, PGJ_2_, PGD_3_/PGE_3_, PGF_3α_, TXB_2_; *5-LOX* – RvE1, RvE2, *trans*-LTB_4_, *epitrans*-LTB_4_, LTB_4_/5*S*,12*S*-diHETE, 5*S*,6*R*-diHETE, 20-OH LTB_4_, LTB_5_, 5-HETE, 5-HEPE, 7-HDHA; *12-LOX* – MaR1, MaR2, 12-HETE, 12-HEPE, 14-HDHA; *15-LOX* – PDx, PD1, RvD1, RvD2, RvD3, RvD4, RvD5, RvE4, LXA_4_, LXB_4_, LXA_5_, 5*S*,15*S*-diHETE, 15-HETE, 15-HEPE, 17-HDHA. Values are given as ng per 50 mg organ. The blue dotted line represents mean levels in adult, group-housed (Ad-GH) mice. LOD of the oxylipins is indicated, if applicable. **e** Radar charts of individual oxylipins in aged, group-housed mice (O-GH, blue) and aged mice undergoing repeated isolation (O-ISO, red). Statistics: Data are shown as (**a**–**d**) mean ± SEM. The number of biological replicates is *n* = 5 for Ad-GH and O-ISO, *n* = 6 for O-GH. One-way ANOVA with post-hoc Šídák’s multiple comparisons test with or without Brown-Forsythe and Welch correction was performed for the indicated comparisons, with # for the comparison of O-GH versus Ad-GH and * for the comparison of O-ISO versus O-GH.

In order to further characterise the age- and isolation-related effects on the oxylipin formation, we classified the oxylipins according to their rate-limiting biosynthetic enzymes, i.e., COX, 5-LOX, 12-LOX, and 15-LOX. In detail, for COX products, age-related increases were most pronounced in the heart (3.71-fold) with minor changes in other organs (Fig. 3a, Supplementary Fig. S2). Isolation did not affect COX products in most organs (fat, heart, lung, and muscle), except for brain (0.71-fold), liver (2.79-fold) and spleen (1.41-fold; Fig. 3a). Taken together, the effect size and its statistical significance indicate that age and isolation only have a moderate impact on COX-derived oxylipins in most organs.

5-LOX products were much more affected by age and isolation. Thus, 5-LOX product levels strongly decreased in fat, liver, muscle, and spleen of aged mice compared to adult controls (Fig. 3b, Supplementary Fig. S2). Conversely, ageing markedly increase 5-LOX products in the heart, which is surprisingly not further aggravated by isolation (Fig. 3b, Supplementary Fig. S2). For all other organs – except muscle where 5-LOX products were not detectable in aged mice – isolation led to a marked increase of 5-LOX-derived oxylipins (Fig. 3b, brain: 2.0-fold, fat: 2.1-fold, liver: 18.2-fold, lung: 8.8-fold, spleen: 17.1-fold). The same trend was observed for oxylipins produced by 12- and 15-LOX (Fig. 3c, d, Supplementary Fig. S2). Thus, ageing caused only moderate changes in the levels of 12-LOX and 15-LOX products, while social isolation caused a dramatic increase for both 12-LOX (brain: 5.7-fold, fat: 7.1-fold, liver: 30.1-fold, lung: 4.5-fold, muscle: 22.2-fold, spleen: 4.3-fold) and 15-LOX products (brain: 1.6-fold, fat: 7.5-fold, liver: 33.4-fold, lung: 7.9-fold, muscle: 2.8-fold, spleen: 15.6-fold) in all organs except for the heart (no elevation). In more detail, the radar charts showing individual representative oxylipins for each enzyme class (COX: PGE_2_; 5-LOX: LTB_4_ and 5-HETE; 12-LOX: 12-HEPE, 12-HETE and 14-HDHA; 15-LOX: PDX, 15-HEPE, 15-HETE and 17-HDHA) confirm the massive elevation of LOX-derived oxylipins in all organs caused by social stress due to isolation, except for heart, with minor or even opposite impact on COX products (e.g., PGE_2_). Together, social isolation stress consistently upregulates LOX-derived oxylipins in organs of aged mice while only the heart and, to a lesser extent, the brain exhibit a lower susceptibility to social stress-related oxylipin alterations (Fig. 3e). These increases in LOX-derived oxylipins were evident for conversion of all PUFAs as substrates, i.e. AA, EPA and DHA.

### Late-life exercise cannot rescue isolation effects on inflammatory state

**Fig. 4:**
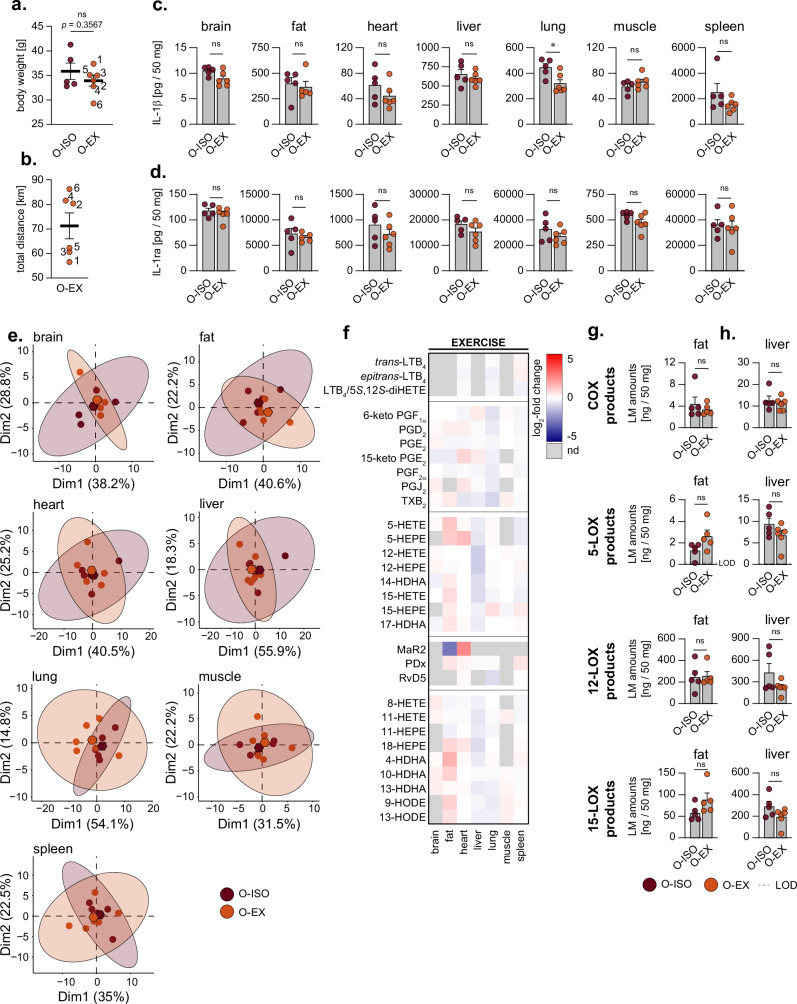
Exercise cannot ameliorate increased oxylipin secretion in aged mice affected by social isolation stress. **a** Body weight of aged (20 month) mice after being repeatedly isolated into single cages with (O-EX) or without running wheels (O-ISO) for 3 separate nights per week over a period of 8 weeks. **b** Total running performance of O-EX mice is summarised in km over the experimental period of eight weeks. **a**, **b** Numbers indicate the individual animals in the O-EX cohort. **c**, **d** Concentration of IL-1β and IL-1ra in pg per 50 mg organ from O-ISO and O-EX mice. **e** Principal component analysis of organ-specific oxylipin profiles from O-ISO and O-EX mice. Hexagons indicate the mean PCA score of all screened replicates within respective experimental groups. **f** Log_2_-fold changes for the organ-specific levels of individual oxylipins comparing the levels of O-EX versus O-ISO mice. Fold-changes that could not be calculated due to missing values (e.g., below limit of detection = nd) are depicted in grey. Total amounts of grouped oxylipin species derived from the COX or LOX pathways in **g** fat and **h** liver. Oxylipins were grouped as indicated in Fig. 3. Values are given as ng per 50 mg organ. Limit of detection (LOD) of oxylipins is indicated, if applicable. Statistics: Data are shown as (**a**–**d**, **g**, **h**) mean ± SEM. The number of biological replicates is *n* = 5 for O-ISO and *n* = 5–6 for O-EX. Unpaired, two-tailed Student’s *t* tests with or without Welch-correction were performed for indicated comparisons.

Since voluntary exercise yields a multitude of positive outcomes prolonging the life and health span^30^ with impact on cytokines and oxylipins^31,32^, we further investigated whether exercise can mitigate the effects on the inflammatory mediators induced by social isolation stress. We employed voluntary wheel running as a late-life exercise intervention in socially isolated aged mice (runners: O-EX, non-runners: O-ISO) by providing running wheels to O-EX mice during the isolated nights of the 8-week-long repeated social isolation period. After the intervention period, the group of O-ISO mice (non-runners) showed a mean weight of 35.9 ± 1.7 g, whereas the group of O-EX mice (runners) showed a slight decrease in body weight (33.9 ± 1.1 g), yet this difference was not significant (Fig. 4a, *p* = 0.357). However, the weight loss seems to be connected to the individual running performance of the mice, as the mouse with the lowest running performance across the 8-week-long experimental period showed the highest weight and vice versa (Fig. 4b).

We then investigated the levels of pro-inflammatory cytokines IL-1β, IL-6, IL-12 p70, TNF-α and anti-inflammatory IL-1ra and assessed the oxylipin signature profiles of organs from aged mice during isolation that were either allowed to exercise (O-EX) or not (O-ISO). Only in the lung, exercise led to a significant decrease in IL-1β levels (0.72-fold) (Fig. 4c) and a small decrease by trend was observed in heart (0.72-fold, *p* = 0.241) and spleen (Fig. 4c, 0.62-fold, *p* = 0.151). For all other organs, the IL-1β levels were essentially unchanged. Although no significant reductions in the other pro-inflammatory cytokines were observed in the organs of O-EX mice, most organs, except for the brain, showed a decrease by trend (Supplementary Fig. S3). In contrast, IL-6 levels in the brain, were significantly elevated. Similarly, IL-1ra levels remained essentially unaltered between O-EX and O-ISO, without significant impact of exercise in any organ (Fig. 4d). Principal component analysis of the overall oxylipin profiles showed no marked differences (no separation of clusters) for all organs comparing O-ISO versus O-EX, with both the position of mean and 95% confidence intervals being similar (Fig. 4e). Yet, when looking at individual oxylipins in detail (Fig. 4f), oxylipin levels in fat (increased) and liver (decreased) were most affected by exercise, particularly pronounced for monohydroxylated fatty acids like 5-HETE, 4-HDHA, 12-HETE, 12-HEPE etc. (Fig. 4f). Interestingly, in the lung, exercise decreased PG and RvD5 formation but did not affect other oxylipins (Fig. 4f). Analysis of oxylipin groups according to biosynthetic enzymes (i.e., COX, 5-, 12-, 15-LOX) reveals no significant alterations due to exercise in any organ (Supplementary Fig. S4), and only in fat and liver a tendency for changes was observed (Fig. 4g, h). Thus, the most notable change by trend upon exercise is the increase of 5- and 15-LOX-derived products in fat tissue (5-LOX: 2.02-fold, *p* = 0.131; 15-LOX: 1.52-fold, *p* = 0.082) while COX- and 12-LOX-derived products remain at a similar level to sedentary mice (Fig. 4g). Furthermore, exercise led to a small decrease in LOX-derived products (5-LOX: 0.72-fold, *p* = 0.214; 12-LOX: 0.52-fold, *p* = 0.140; 15-LOX: 0.67-fold, *p* = 0.188) in the liver, while the level of COX-derived products was unaltered (Fig. 4h). Together, for aged mice undergoing social isolation stress, the variable of voluntary exercise has only a minor impact on the massive alterations of oxylipins and cytokine levels caused by isolation.

### Cognitive function does not benefit from voluntary exercise in isolation

In a previous study^27^, we addressed the relation between age and cognition in male, group-housed mice by subjecting them to a Barnes maze test, revealing a cognitive decline that occurs between the ages of 15 and 24 months. It was also shown that for aged mice, constant social isolation further impairs cognitive function. To evaluate whether voluntary wheel running can mitigate cognitive decline in ageing and social stress through repeated isolation and resocialisation, we tested isolated aged mice in the Barnes maze to assess spatial learning, as well as short- and long-term memory. The timeline of the implementation of the Barnes maze test is shown in the schematic in Fig. 1. During the training period for the Barnes maze test, both cohorts with recurring social stress (O-ISO and O-EX) showed a similar, improved performance over time.

**Fig. 5:**
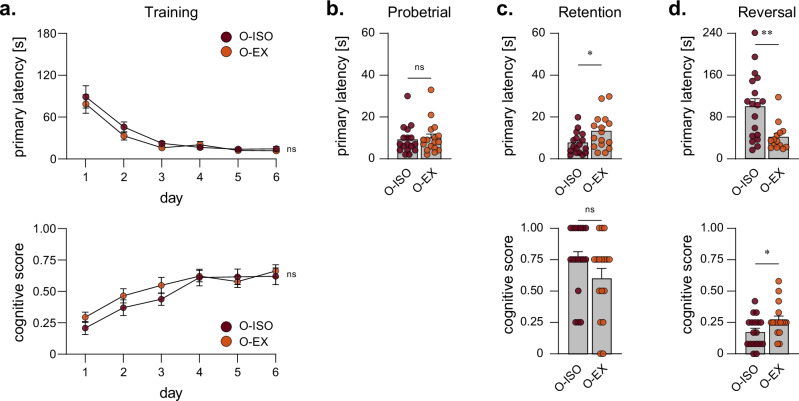
Cognitive function assessment of mice undergoing recurring social isolation with and without physical exercise. Results from the Barnes maze test, depicted as the primary latency period and as cognitive scores for **a** training, **b** probe trial, **c** after a retention period, and **d** in a reversal test. Statistics: Data are shown as mean ± SEM. The number of biological replicates is *n* = 18 for O-ISO and *n* = 17 for O-EX. Unpaired, two-tailed Student’s *t* tests with or without Welch-correction were performed for indicated comparisons.

No strong exercise-mediated improvements were observed during training (Fig. 5a). The probe trial (short-term memory) and the retention test (long-term memory) did not reveal cognitive improvement following exercise, but instead a prolonged primary latency in the retention test of the O-EX group (Fig. 5b, c). During the reversal test (cognitive flexibility), the O-EX mice showed a shorter primary latency and an increased cognitive score compared to O-ISO mice, suggesting a potential facilitation in task adaptation (Fig. 5d). Overall, exercise intervention in late life does not seem to improve spatial learning, short and long-term memory in aged mice experiencing social isolation stress, but O-EX showed an improved cognitive flexibility.

### Reduced age-associated inflammatory repertoire of oxylipins and proteins in serum is maintained after isolation and exercise

**Fig. 6:**
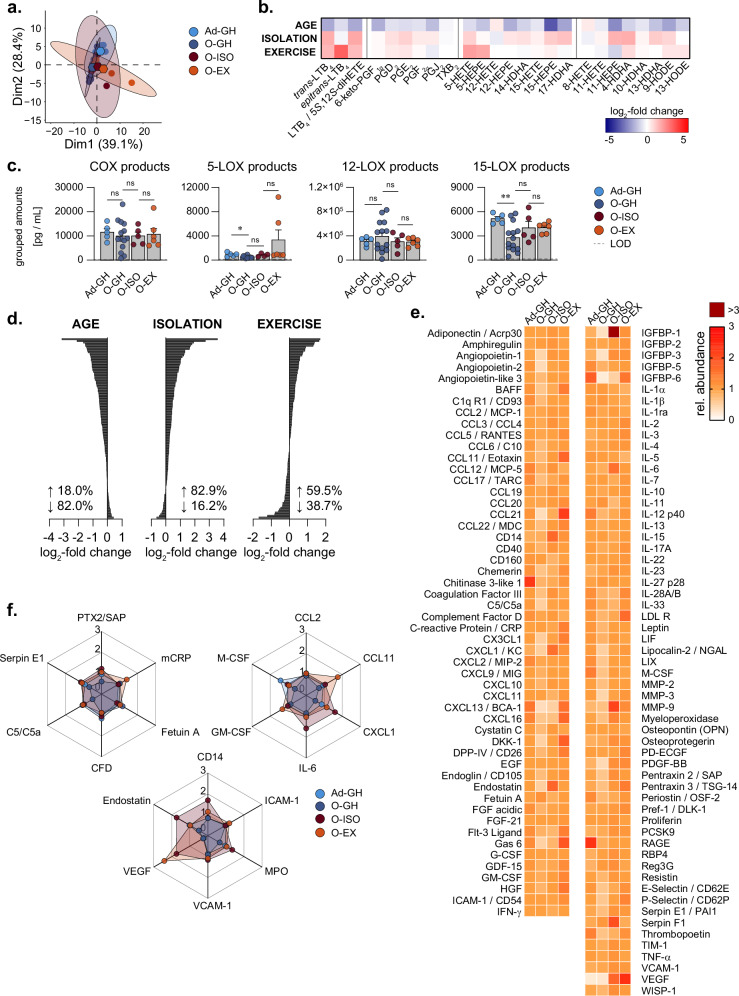
Impact of age and social isolation on inflammatory oxylipins and proteins in serum. **a** Principal component analysis of oxylipin profile of serum. Hexagons indicate the mean PCA score of all screened replicates within respective experimental groups. **b** Log_2_-fold changes for the concentrations of individual lipid mediators, detected in serum, comparing the levels of (AGE) O-GH versus Ad-GH, (ISOLATION) O-ISO versus O-GH, and (EXERCISE) O-EX versus O-ISO mice. **c** Total amounts of grouped LM species that are products of the COX or LOX pathways in serum. Metabolites were grouped as indicated for Fig. 3. Values are given as pg per mL serum. Limit of detection (LOD) of the metabolites is indicated, if applicable. **d** Log_2_-fold changes of 111 screened, circulating proteins in pooled serum samples and percentages of regulated proteins for the depicted comparisons. **e** Heatmap showing the relative abundance of the investigated proteins in all experimental cohorts. **f** Radar charts displaying the relative abundance of selected (top left) acute-phase proteins, (top right) classical cytokines and chemokines, and (bottom) various surface markers and inflammatory proteins. Statistics: Data are shown as mean ± SEM (**c**). The number of biological replicates is *n* = 5 for Ad-GH and O-ISO, *n* = 14 for O-GH and *n* = 6 for O-EX. **d**–**f** Samples were pooled from the serum of 4–5 biological replicates for all screened cohorts. One-way ANOVA with post-hoc Šídák’s multiple comparisons test with or without Brown-Forsythe and Welch correction was performed for the indicated comparisons.

To determine whether the observed organ-specific effects due to ageing and isolation as well as exercise are reflected in the systemic circulation, we characterised the inflammatory secretome (oxylipins and proteins) in the serum of the mice cohorts from above. Based on both, the principal component analysis of the oxylipin profiles and the detailed analysis of the individual oxylipins, age but not isolation or exercise is the main driver for the observed alterations (Fig. 6a, b). While in the PCA all aged cohorts (O-GH, O-ISO, O-EX) cluster tightly together, the oxylipin profile of the adult cohort (Ad-GH) shows a distinct shift (Fig. 6a). The majority of the screened oxylipins in serum are decreased due to age (O-GH/Ad-GH, Fig. 6b) with significant changes for 5- and 15-LOX-derived products (5-LOX: 0.55-fold, 15-LOX: 0.54-fold, Fig. 6c). To our surprise and in contrast to organs (see above), isolation only had a minor impact on oxylipin levels in serum, reflected by the lack of changes for COX and 12-LOX products, and only moderate increases in 5- and 15-LOX-derived products in serum from O-ISO versus O-GH mice (5-LOX: 1.63-fold, *p* = 0.087; 15-LOX: 1.44-fold, *p* = 0.250, Fig. 6c). These serum oxylipin levels of O-ISO did not markedly change due to exercise, apart from a 4-fold increase in 5-LOX-derived products (O-ISO: mean = 822.2 ± 124.0 pg/mL, O-EX: mean = 3341.3 ± 1661.3 pg/mL; *p* = 0.350) which was, however, not significant and attributed to only 2 out of the 6 biological replicates (Fig. 6c).

We next employed an unbiased analysis of the repertoire of inflammation-related cytokines, chemokines, enzymes, and growth factors in the serum of Ad-GH, O-GH, O-ISO, and O-EX mice, using a commercially available Proteome Profiler, allowing us to screen for the relative abundance of 111 candidates. In line with the reduced amounts of most of the oxylipins (Fig. 6b), ageing led to an overall downregulation (82.0%) of the investigated proteins in serum (Fig. 6d). Isolation, however, led to an increased expression of 82.9% of the proteins (Fig. 6d). Despite the seemingly contradictory dynamics, the proportions of proteins being down-regulated with age and up-regulated due to isolation are not congruent (Supplementary Fig. S5d). The exercise intervention (O-EX) led to less pronounced and more balanced alterations for aged mice under isolation (O-ISO) with 59.5% up- and 38.7% downregulated proteins (Fig. 6d). Acute phase proteins (APPs, e.g., complement factors and pentraxins) and chemokines (particularly of the CCL and CXCL classes) were impaired due to age versus adult mice (Fig. 6e, f). Such overarching class effects could not be observed for the cohorts O-ISO and O-EX under isolation. Interestingly, the pro-inflammatory cytokine IL-6, the chemokine CXCL1, and the lipopolysaccharide (LPS) receptor cluster of differentiation (CD)14 were increased in O-ISO versus O-GH mice (Fig. 6e, f). Exercise of aged mice under isolation, however, induced higher levels of murine C-reactive protein (CRP), CCL11, CCL21, and vascular endothelial growth factor (VEGF) versus O-ISO mice (Fig. 6e, f). These alterations in the abundance of inflammation-related serum markers indicate a reduced immune competence with age, and distinct pro-inflammatory responses due to isolation and exercise.

## Discussion

Social isolation, a phenomenon commonly occurring in late life, is extensively described as a risk factor for increased morbidity and mortality and is associated with higher levels of inflammation and cognitive decline^36,37^. Considering inflammation as a shared feature of ageing^38^, isolation^39,40^, and an inactive lifestyle^41^, we examined the inflammatory cytokine and oxylipin secretome in mice undergoing recurring social isolation and resocialisation, and whether voluntary physical exercise can ameliorate the inflammatory stress responses due to isolation in single housing. Unexpectedly, we found dramatic elevation of inflammation-related LOX-derived oxylipins due to social isolation in an organ-specific manner, especially affecting liver, lung, and spleen. Surprisingly, physical exercise showed a neglectable impact on the organ-intrinsic oxylipin secretome of isolated mice and was hardly evident in systemic circulation.

Previous studies have frequently investigated and described social isolation as a stressor, resulting in a variety of adverse health outcomes^42,43^. The magnitude of these events, however, varies greatly depending on the type and duration of social isolation^44,45^. Studies on permanent or short- to long-term social isolation reported mixed and sometimes contradictory results. Thus, a four-week social isolation period of both male and female adult mice (3 months) affected female animals more than males, but overall, the isolation only had minimal effects on serum levels of the stress marker corticosterone, behaviour, and body weight^46^. Conversely, shorter periods of single housing of mice, such as 4–5 days only, tend to produce more pronounced detrimental effects, including anxiety behaviour, elevated urinary stress hormones, and weight loss^28^. These findings suggest that, while isolation quickly triggers a strong stress response, mice may adapt within a few weeks, resulting in lower or unchanged stress markers over prolonged isolation. Nevertheless, despite seemingly modest stress responses, long-term isolation of degus over a period of 20–24 months still promotes a pro-inflammatory state^47^. Research on the effects of long-term isolation in human cohorts is limited. One study reported elevated cortisol levels and heightened immune activation in men after an isolation period of ~1.5 years^48^. However, to allow comparability between studies on isolation, further adjustments to the experimental conditions are needed. In our study, aged mice underwent a social stress protocol of repeated isolation in single housing for three individual nights per week and subsequent resocialisation in group housing over a total period of 8 weeks. To allow for robust comparisons of the inflammatory markers following the isolation and exercise intervention against a robust baseline, we considered a group-housed control cohort at 5 months of age as a fully mature cohort, in which most biological and behavioural processes have largely stabilised, to best reflect physiological and healthy conditions. Since social isolation in humans is particularly an increasing problem in elderly communities and healthy young adults have a far lower prevalence for it^49,50^, we deliberately opted against the implementation of an adult cohort that undergoes the isolation protocol.

Our results confirm that repeated social isolation acts as a potent and impactful stressor, elevating several pro-inflammatory cytokines. In previous studies, it was proven that sympathetic signalling can induce a pro-inflammatory state in myeloid cells that is characterised by enhanced IL-1β, IL-6 and TNF-α gene expression^51,52^. Similarly, social stress conditions may induce a systemic and local tissue-specific activation of the immune system through NF-κB signalling, which in turn leads to increased cytokine formation. We showed before, that oxylipins are affected by ageing in an organ-specific manner^17^, which we confirm with similar effect sizes for the comparison of mature adult (5 months) and aged mice (20 months) in this study. In agreement with this previous study, we did not find a systemic upregulation of pro-inflammatory cytokine and oxylipin signalling across several organs, further challenging the concept of a universal pro-inflammatory microenvironment as the primary phenotypic trait of inflammaging. Additionally, we here uncover a dramatic upregulation of LOX-derived oxylipin production in several organs due to isolation, while COX products that are produced from the same PUFA substrates^34^ were hardly altered. Increased IL-1β has previously been linked to an isolation-associated inflammatory phenotype^53^, but our findings demonstrate, for the first time the tremendous effect of social isolation stress on oxylipins as responsible mediators of the inflammatory response in several organs. Since pro-inflammatory cytokines (i.e. IL-1β, IL-6, TNF-α, IL-12) and oxylipins are tightly intertwined in a bidirectional inflammatory network^54^, we believe that an assessment of both biomarker classes is imperative to assess the immunological consequences of social isolation. Since long-term elevation of monohydroxylated LOX-derived oxylipins and pro-inflammatory cytokines have been linked to tissue fibrosis and organ damage^55–58^, we suspect that the elevated inflammatory secretome after repeated social isolation may aggravate organ-specific inflammaging and the development of age-associated malignancies. Notably, these age- and isolation-related effects did not occur systemically, reflected by the overall moderate changes of oxylipin and cytokine levels in serum of mice. Hence, biomarker profiles in serum cannot be considered a suitable surrogate to elucidate the organ-specific inflammatory microenvironment shaped by cytokines and oxylipins.

The dramatic changes in oxylipin formation due to isolation may be attributed to a dysregulation of the hypothalamic-pituitary-adrenal (HPA) axis in response to social isolation as a psychosocial stressor. It is known that oxylipins, particularly eicosanoids such as PGs, can be affected by stress^59^, and that stress may influence the expression of COX-1 and -2^60,61^. LOX expression has likewise been shown to be regulated through stress signalling, which can decrease the pro-inflammatory immune response along with elevated 15-LOX-2 expression and elevated SPM levels^62,63^. This could explain the observed increase in 15-LOX-derived products. Our present results documenting elevated LOX products in several organs and tissues of aged mice due to social isolation stress, predominantly in immunological organs like spleen, liver, and lung, are in good agreement with the above-mentioned impact of stress on oxylipin biosynthesis. Whether or not isolation of aged mice elevates LOX expression remains to be explored in more detail.

Aberrations in inflammatory signalling caused by age and isolation may raise the risk of dysregulated homeostasis, leading to chronic basal inflammation. Cognitive decline may also result from age- and stress-related imbalances in oxylipins, as PG signalling and 5-LOX expression have been associated with reduced cognitive function and depression-like symptoms^64–66^. Furthermore, chronic social stress has been found to significantly elevate levels of 12-LOX-derived metabolites 12-HETE and 12-HEPE in the brain^67^. In our study, isolation of aged mice elevated both, 5-LOX products with mainly pro-inflammatory features^68^ but also 12/15-LOX-derived SPM like RvD5, PDX, and MaR2 and their precursors 17-HDHA and 14-HDHA, which act as immunoresolvents that terminate and resolve inflammation^10^. Therefore, these alterations in oxylipin profiles may be connected to a stress-induced activation of the innate immune system, triggering the mobilisation of neutrophils, monocytes, and monocyte-derived macrophages, as primary producers of oxylipins^69^.

Lifestyle interventions such as diet or exercise are popular and well-characterised means of mitigating or circumventing negative age-related health outcomes^70,71^. Exercise has been shown to exhibit anti-inflammatory features, which are, however, highly dependent on type, duration, onset, and intensity^72^. For example, Sun et al. found that exercise indeed reduces inflammageing features like IL-1β secretion during long-term exercise (12-month voluntary exercise) in young and aged mice exposed to infectious injury^73^. Thus, mice that underwent exercise were more protected from LPS challenge than sedentary mice. However, this effect was less pronounced in mice that started the exercise regimen at an older age^73^, and is in line with results indicating that adaptive and acute responses to physical activity decline with advancing age^74^. This may also explain why our results did not confirm previous findings showing that exercise stimulates both pro- as well as anti-inflammatory pro-resolving lipid mediators^32,75,76^. In contrast to these studies, our exercise protocol started in late life (18 mo), in addition to the stress factor of repeated social isolation and resocialisation. We suppose that the detrimental effects of social isolation override the benefits of physical exercise, and thus, no ameliorating effect of exercise on pro-inflammatory signatures could be observed.

While our protocol of repeated isolation and resocialisation may better mimic isolation in the elderly than current long-term isolation models, several limitations should be recognised. Past studies have shown that the effects of social isolation occur in a sex-specific manner, with female mice generally being more affected than males^46^. Additionally, initiating the exercise in late life may have limited its potential benefits as the adaptive capacity of aged organisms is reduced^77^. Although it would be valuable to determine whether an earlier start of the exercise intervention could mitigate or even prevent negative social isolation effects, we deliberately chose to evaluate exercise as a direct countermeasure to already established social isolation. This approach was intended to better reflect real-life scenarios in which interventions are implemented after negative outcomes occurred. Lastly, our study does not address whether the detrimental effects of repeated social isolation represent a persistent phenotype or occur as a transient state that may be reversed through sustained social reintegration.

In future studies, the molecular signalling circuits that connect social isolation and inflammageing need to be further explored, particularly the bidirectional interplay of LOX-derived oxylipins and inflammatory cytokines. It is also important to recognise that the idea of gradual ageing is increasingly challenged, with findings indicating that nonlinear ageing may be more common and that abrupt changes might amplify ageing processes^78–80^. As such, social isolation stress in elderly may push individuals past a critical threshold, triggering escalation of inflammation and accelerating ageing. Hence, it will become particularly relevant to study the impact of social isolation stress in the context of common age-associated infectious conditions^81,82^, which would allow to assess functional immune competence and immunological resilience in ageing.

Our findings demonstrate that repeated cycles of social isolation and resocialisation may act as stressors, increasing inflammation-related cytokine secretion and dramatically enhancing the formation of LOX-derived oxylipins in defined organs, impacting inflammation and tissue homeostasis. Notably and to the best of our knowledge, this is the first report revealing organ-specific oxylipin modulation upon social isolation, offering a novel framework for future interdisciplinary research at the intersection of inflammation, ageing, and social isolation. Under our experimental conditions, voluntary physical exercise did not counteract the consequences of social isolation stress on oxylipin production. Conclusively, the dramatic elevation of LOX-derived oxylipins highlights the impact of social isolation stress, a common phenomenon in the elderly, on inflammatory mediators within several specific organs and propose oxylipins as determinants of stress-related inflammageing.

## Methods

### Animals and sample isolation

All animal procedures were performed according to the ARRIVE guidelines and the German Law on the Protection of Animals/European Communities Council Directive (86/609/EEC). Animal experiments were approved by the Thuringia State Office for Food Safety and Consumer Protection (licenses UKJ-19-014 and TWZ09-2022).

Male C57BL/6J/UKJ mice were bred and housed at the Central Experimental Animal Facility at Jena University Hospital, Jena, Germany. Mice were kept on a 14/10 h light/dark cycle and at 22 ± 2 °C with a relative humidity of 55 ± 10%. The mice had unrestricted access to water and food (ssniff mouse V1534-300, ssniff Spezialdiäten GmbH, Soest, Germany, a detailed overview on the composition can be accessed via the manufacturer).

For all animal experiments, adult (5 months of age) and old (20 months of age) mice were randomly assigned to the following four experimental cohorts: (1) Ad-GH (*n* = 5), adult mice that were kept in group-housing throughout their lives; (2) O-GH (*n* = 6), 20-months-old mice that were kept in group-housing throughout their lives; (3) O-ISO (*n* = 5 for oxylipin analysis, *n* = 18 for cognitive testing), aged mice that were kept in group-housing until 18 months of age, then over a period of 8 weeks, repeatedly separated for three individual nights per week (Sunday, Tuesday, Thursday) into single cages before being returned to their littermates; and (4) O-EX (*n* = 6 for oxylipin analysis, *n* = 17 for cognitive testing), aged mice treated identically to O-ISO mice but provided with running wheel during the isolation nights, allowing for voluntary wheel running. Estimation of necessary animal numbers per experiment was based on previous animal applications, long-standing animal testing practice and by statistical plausibility criteria.

To minimize stress, experimental procedures (social isolation and wheel running) were conducted in the same room where animals were kept and simultaneously for all experimental groups whenever possible. Behavioural assessment was performed in another room after an initial acclimation phase of 30 min before each test.

After the experimental period, mice were euthanised by cervical dislocation without prior anaesthesia. Whole blood was collected immediately after death. To extract serum, blood was kept at room temperature for around 60 min to ensure clotting. Samples were subsequently centrifuged for 30 min at 2000 × *g*. Serum was transferred into fresh reagent tubes and stored at −80 °C. Seven organs, namely brain, fat (visceral white adipose tissue), heart, liver, lung, muscle (quadriceps and gastrocnemius), and spleen were harvested, washed with PBS (pH 7.4, SERVA, Heidelberg, Germany; 47302.03), and stored at −80 °C.

### Cognitive testing with Barnes maze

The Barnes maze is employed to assess spatial learning, short- and long-term memory, memory retrieval, and cognitive flexibility by training animals to associate distal cues with a fixed escape box^27^. Mice are placed on a brightly lit circular platform with 20 holes, one of which provides access to an escape box. The protocol comprises one day of habituation, followed by 6 training days (3 trials/day with an inter-trial interval of 60 min) to assess learning. The following day, a probe trial, for which all holes are closed, evaluates short-term memory. After three days without further training, a retention test assesses long-term memory retention with the original hole with the escape box opened again. Lastly, one day later the position of the escape box is placed 180° opposite to the original position. This reversal test measures cognitive flexibility.

Animals that displayed a primary latency higher than twice the standard deviation of the respective group (O-ISO: *n* = 1, O-EX: *n* = 2) were excluded from further analysis within in this study.

### Organ homogenisation

For fat, liver, lung, muscle and spleen, organ homogenates were prepared by weighing 20–40 mg of organ tissue in tubes containing around 100 mg of lysing matrix D per 10 mg organ (M.P. Biomedicals, Irvine, CA, USA; 116540434). The lysis buffer was added at a ratio of 20 µL per milligram organ (1% (*v*/*v*) NP-40 (AppliChem, Darmstadt, Germany; A1694), 1 mM sodium orthovanadate (AppliChem; A2196), 10 mM sodium fluoride (AppliChem; A3904), 5 mM sodium pyrophosphate (Sigma Aldrich, St. Louis, MO, USA; S8282), 25 mM β-glycerophosphate (Sigma Aldrich; G9422), 5 mM EDTA (AppliChem; A2937), 25 µM leupeptin (Sigma Aldrich; L2884), 3 mM soybean trypsin inhibitor (Sigma Aldrich; T9128) and 1 mM phenylmethanesulfonyl fluoride (Sigma Aldrich; P7626)). For the homogenisation of brain and heart, one hemisphere and the whole heart, respectively, were weighed into tubes containing lysing matrix D, and 5 µL of lysis buffer per milligram organ was added. All organs were homogenised using a FastPrep-24™ 5 G bead beating homogeniser using established protocols (M.P. Biomedicals).

### Cytokine quantification via ELISA

For cytokine quantification, homogenates were centrifuged at 21,100 × *g*, 4 °C for 10 min. The supernatant was transferred into fresh reagent tubes and stored at −20 °C until further analysis. The final concentration of the homogenates was 50 mg/mL for fat, liver, lung, muscle, spleen, and 200 mg/mL for brain and heart.

Quantification of cytokines in organ homogenates was performed using commercially available ELISA kits for murine IL-1β (DY401-05), IL-1ra (DY480), IL-6 (DY406-05), IL-12 p70 (DY419-05), and TNF-α (DY410-05) manufactured by R&D Systems (Minneapolis, MN, USA). The assays were completed per the manufacturer’s instructions. Quantification was based on standard curves generated individually for each experiment. Values that could not be computed were set to 0 for downstream analyses.

### Oxylipin quantification via UPLC-tandem mass spectrometry

For oxylipin quantification, immediately after homogenisation, homogenates were mixed 1:1 with ice-cold methanol (fisher chemical; 10653963). Samples were kept on ice for around 20 min, and then centrifuged at 21,100 × *g*, 4 °C for 10 min. The amount of supernatant equivalent to 20–30 mg organ was then transferred into glass vials containing 990 µL ice-cold methanol and 10 µL of deuterium-labelled internal standard [200 nM *d*_8_-5*S*-hydroxyeicosatetraenoic acid (HETE) (Cayman Chemical; 334230), *d*_4_-LTB_4_ (Cayman Chemical; 320110), *d*_5_-LXA_4_ (Cayman Chemical; 24936), *d*_5_-RvD2 (Cayman Chemical; 11184), *d*_4_-PGE_2_ (Cayman Chemical; 10007273) and 10 μM *d*_8_-AA (Cayman Chemical; 390010). Finally, lysis buffer was added to yield a total volume of 3 mL and stored overnight at −20 °C to ensure protein precipitation.

After centrifugation (1200 × *g*, 4 °C for 10 min), supernatants were transferred to clean glass vials and acidified by addition of 9 mL of MilliQ water (pH 3.5). Solid phase extraction of organ samples was performed as previously described^83^. Purified samples were evaporated under continuous N_2_ flow, and the residue was resuspended in 200 µL of an equal mixture of methanol and water (VWR Chemicals, 83645320). After final centrifugation at 21,100 × *g*, 4 °C for 5 min, the supernatant was used for UPLC-MS/MS analysis. Analytes were separated on an Acquity UPLC system (Waters, Milford, MA, USA) equipped with an Acquity UPLC BEH C18 column (1.7 μm, 2.1 mm × 100 mm; Waters, Eschborn, Germany) and a pre-column of identical material with column temperature set to 50 °C. The gradient was adjusted as listed in Table 1. Analytes were detected using a QTRAP 5500 mass spectrometer (ABSciex, Darmstadt, Germany) with electrospray ionisation, operated as previously described^83^.

**Table 1:** Composition of mobile phase and elution gradient for targeted lipidomics

Time [min]	Flow rate [mL/min]	Eluent A [%] (H_2_O/MeOH 90/10 + 0.01% CH_3_COOH)	Eluent B [%] (MeOH + 0.01% CH_3_COOH)
0	0.3	64.4	35.6
4.8–19.0	0.3	45.5	54.5
26.7	0.3	15.6	84.4
29.7	0.3	2.2	97.8
30.7	0.3	64.4	35.6

The QTRAP 5500 was operated in negative mode using scheduled multiple reaction monitoring (MRM) with the MRM window set to 60 s. The retention time and constitutional stereochemistry of analytes were confirmed by external standards (Cayman Chemical), and individual calibration curves were obtained for quantification, using the ratios of the areas of external standards to their corresponding internal standard (ES/IS). Additional information, such as the specific transitions, detection and quantitation limits for each screened metabolite, is listed in Supplementary Data S1. Representative unsmoothed chromatograms from samples showing peaks of oxylipins relevant to this study are provided in Supplementary Fig. S6. For further analysis, the determined concentration was normalised to pg or ng oxylipin per 50 mg organ. The normalised raw data is provided in Supplementary Data S2.

### Assessment of serum proteome (proteome profiler)

Serum samples were pooled from 4 to 5 biological replicates to a final volume of 100 µL. The serum samples were assessed for the relative abundance of 111 cytokines using the commercially available Proteome Profiler Mouse XL Cytokine Array Kit (ARY028) by R&D Systems, following the manufacturer’s guidelines. After treatment with IRDye 800CW streptavidin (LI-COR, Bad Homburg, Germany), and the membranes were analysed with an LI-COR Odyssey Infrared Imaging System. Pictures of the raw blots are included in Supplementary Fig. S5a–c.

### Data handling and statistical analysis

Results are presented as mean ± standard error of mean (SEM) unless stated otherwise. Data analysis was not performed in a blinded mode for all operators. For data analysis and visualisation GraphPad Prism (Version 10.4.1), Origin Pro (Version 2023b), RStudio (2024.12.0) and Adobe Illustrator (version 29.3) were used. For the analysis of the oxylipin data, values below the determined limits of detection (LOD) were set to the corresponding LOD, and values between LOD and the lower limit of quantification (LLOQ) were set to ½ LLOQ, unless stated otherwise. For the principal component analysis, RStudio in combination with the R packages FactoMineR (https://cran.r-project.org/web/packages/FactoMineR/index.html) and factoextra (https://cran.r-project.org/web/packages/factoextra/index.html) was used, and metabolites for which all values were below the LLOQ were excluded from analysis. The ROUT outlier test was used to identify outliers within data sets, with a *Q*-value set to 0.5 (for biomarker data) or 0.1 (for behavioural analysis). Detected outliers were excluded from further analysis. The distribution of datasets was checked by Shapiro–Wilk test with α set to 0.05 and, if implied, data was log-transformed for statistical analysis. For group comparisons, an unpaired, two-tailed Student’s *t* test or one-way ANOVA with post-hoc Šídák’s multiple comparison test was performed as indicated in the figure legends. Statistical significance was assumed for comparisons with *p* ≤ 0.05. Significance is indicated as: *p ≤ 0.05, **p ≤ 0.01, ***p ≤ 0.001, ****p ≤ 0.0001, or # respectively, ns, not significant.

## Supplementary information

Supplementary_Wichmann-Costaganna et.

Data S1 - Screened PUFA metabolites, transitions, LOD and LLOQ.

Data S2 - Mass spectrometry (lipidomic) raw data.

## Data Availability

The datasets analysed during the current study are included in this published article and its supplementary information files.
