# Supplementary material for: Social isolation of aged mice drives dramatic release of inflammatory lipoxygenase-derived oxylipins
Source: NPJ Aging. 2026 May 16;12(1):67. doi: 10.1038/s41514-026-00405-6 (PMC13179954; doi:10.1038/s41514-026-00405-6)
Supplement: Supplementary file 1 — Supplementary_Wichmann-Costaganna et. [file 41514_2026_405_MOESM1_ESM.pdf]

Supplementary Information

Social isolation of aged mice drives dramatic release of inflammatory lipoxygenase-derived oxylipins

Mareike Wichmann-Costaganna[1], Raphaëlle Petit[2], Julia Lindner[2,3], Madlen Haase[2], Vivien Bachmann[1], Robert Klaus Hofstetter[1], Markus Werner[1], Christiane Frahm[2,#], Oliver Werz[1,#], Patrick Schädel[1,#]

[1]Department of Pharmaceutical/Medicinal Chemistry, Institute of Pharmacy, Friedrich Schiller University Jena, 07743 Jena, Germany

[2]Department of Neurology, Jena University Hospital, Am Klinikum 1, 07747 Jena, Germany

[3]Department of Hematology, Cellular Therapy, Hemostaseology and Infectious Diseases, University Hospital Leipzig, University of Leipzig Medical Center and Comprehensive Cancer Center Central Germany (CCCG) Leipzig-Jena, 04103 Leipzig, Germany

[#] shared correspondence

Figure S1: Age- and isolation-induced cytokine formation.

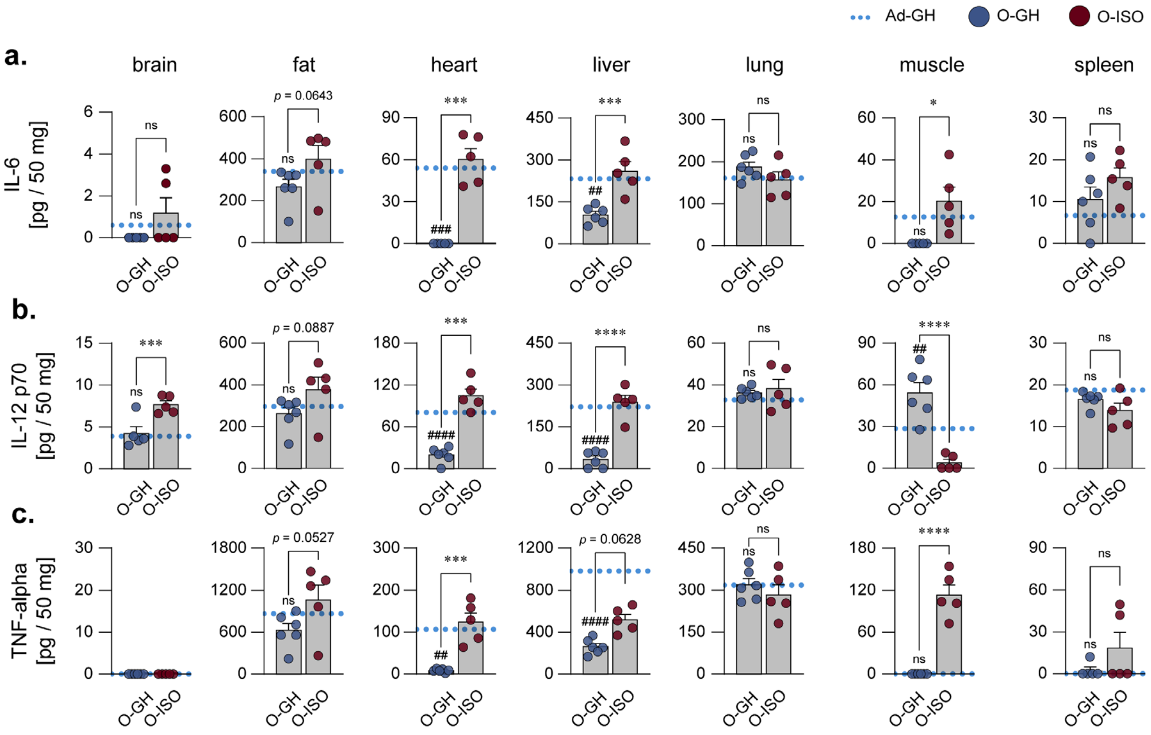

Fig. S1: Age- and isolation-induced cytokine formation. (a-c) Concentration of the cytokines IL-6 (**a.**), IL-12 p70 (**b.**), and TNF-α (**c.**) in pg per 50 mg organ. The blue dotted line represents mean levels in adult, group-housed (Ad-GH) mice. Values that could not be computed were set to 0. **Statistics**: Data are shown as mean ± SEM. The number of biological replicates is *n* = 5 for Ad-GH and O-ISO, *n* = 6 for O-GH. One-way ANOVA with post-hoc Šídák's multiple comparisons test with or without Brown-Forsythe and Welch correction was performed for the indicated comparisons, with # for the comparison of O-GH versus Ad-GH and * for the comparison of O-ISO versus O-GH.

Figure S2: Age-specific oxylipin changes.

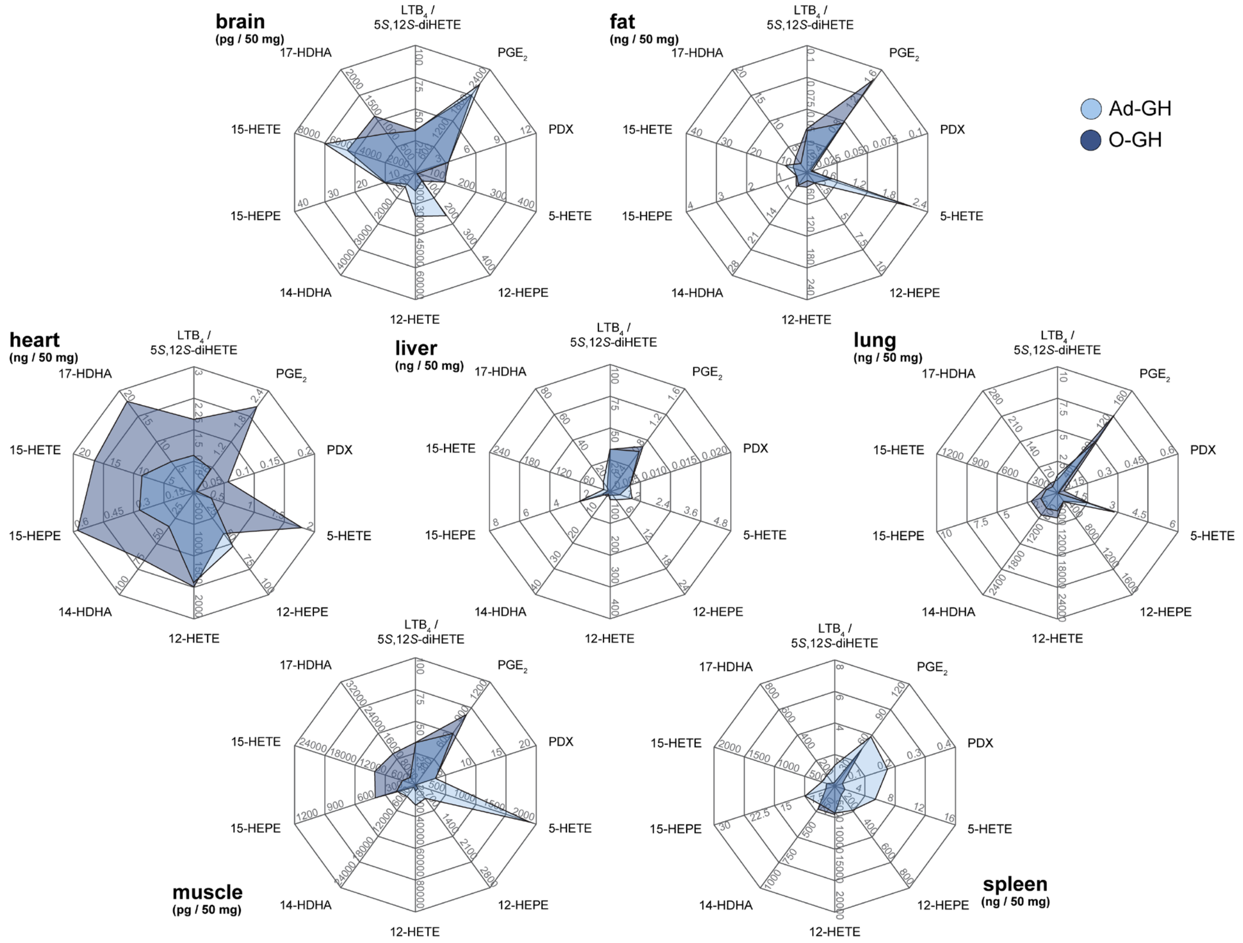

Fig. S2: Age-specific oxylipin changes. Radar charts of individual oxylipins in adult, group-housed mice (Ad-GH, bright blue) and aged, group-housed mice (O-GH, dark blue) in pg (brain, muscle) or ng (fat, heart, liver, lung, spleen) per 50 mg organ. Data are shown as mean.

Figure S3: Exercise-induced cytokine formation.

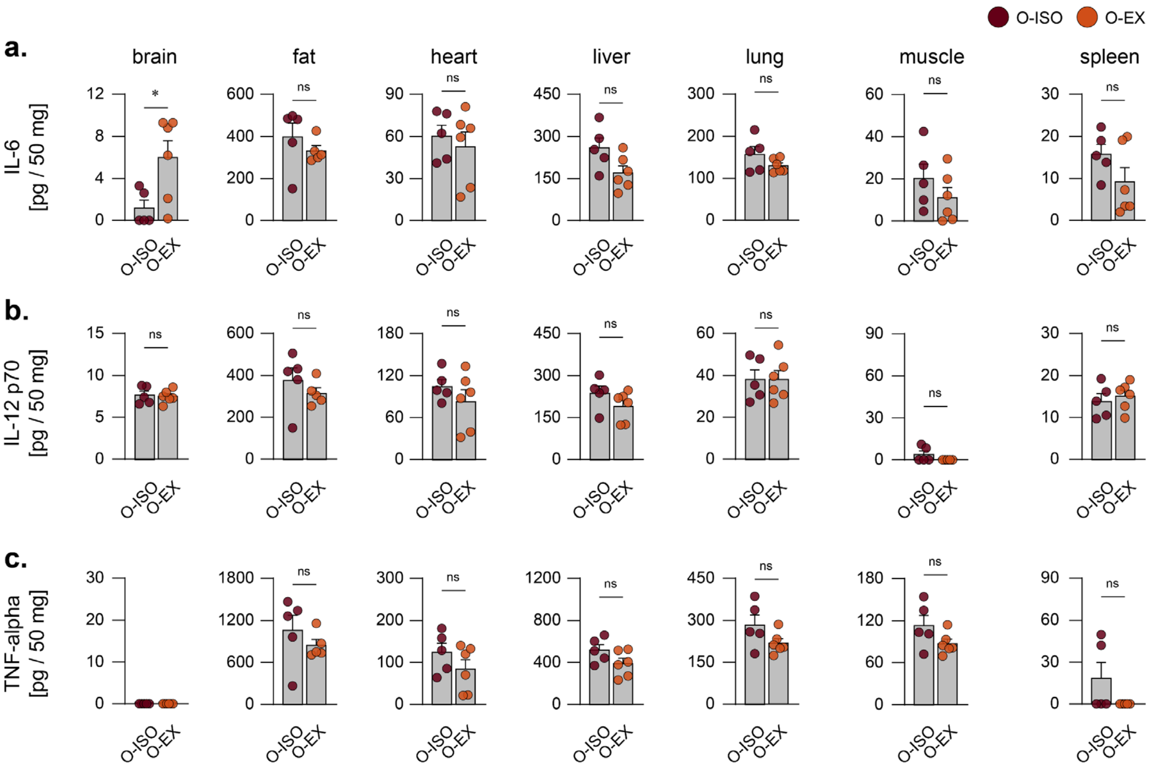

Fig. S3: Exercise-induced cytokine formation. (a-c) Concentration of the cytokines IL-6 (**a.**), IL-12 p70 (**b.**), and TNF-α (**c.**) in pg per 50 mg organ. Values that could not be computed were set to 0. **Statistics**: Data are shown as mean ± SEM. The number of biological replicates is n = 5 for O-ISO and n = 5-6 for O-EX. Unpaired, two-tailed Student's t-tests with or without Welch-correction were performed for indicated comparisons.

Figure S4: Enzyme-derived oxylipin levels for isolated mice with and without exercise.

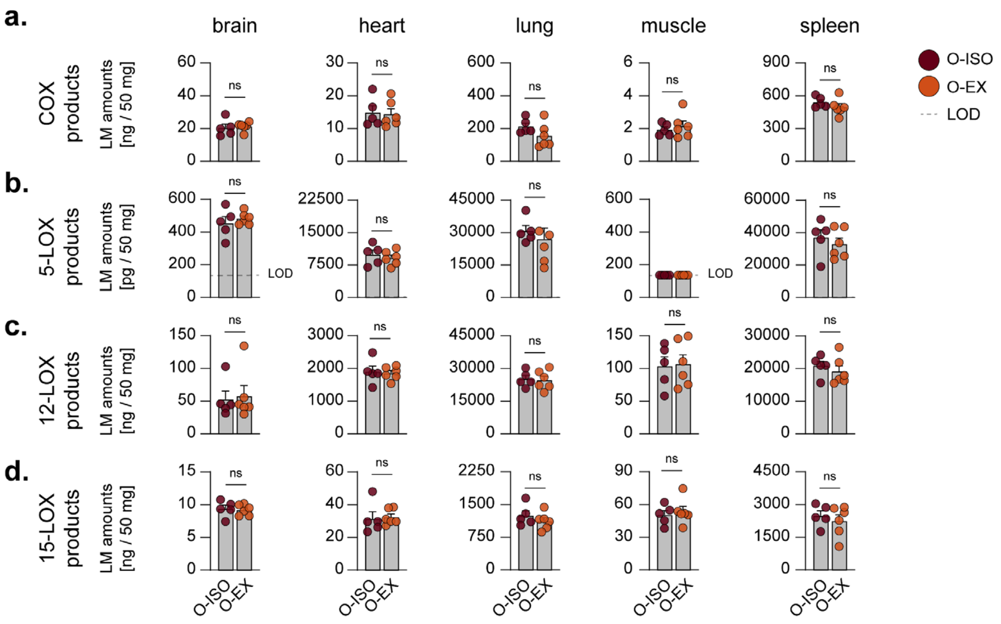

(a-d) Total amounts of grouped LM species for isolated mice with (O-EX) and without exercise (O-GH) that are products of the COX or LOX pathways in brain, heart, lung, muscle, spleen. Metabolites were grouped as follows: **(a)** *COX* – PGD$_1$, PGE$_1$, PGF$_{1\alpha}$, 6-keto PGF$_{1\alpha}$, PGD$_2$, PGE$_2$, 15-keto PGE$_2$, PGF$_{2\alpha}$, PGF$_{2ß}$, PGJ$_2$, PGD$_3$/PGE$_3$, PGF$_{3\alpha}$, TXB$_2$; **(b)** *5-LOX* – RvE1, RvE2, *trans*-LTB$_4$, *epitrans*-LTB$_4$, LTB$_4$/5*S*,12*S*-diHETE, 5*S*,6*R*-diHETE, 20-OH LTB$_4$, LTB$_5$, 5-HETE, 5-HEPE, 7-HDHA; **(c)** *12-LOX* – MaR1, MaR2, 12-HETE, 12-HEPE, 14-HDHA; **(d)** *15-LOX* –PDx, PD1, RvD1, RvD2, RvD3, RvD4, RvD5, RvE4, LXA$_4$, LXB$_4$, LXA$_5$, 5*S*,15*S*-diHETE, 15-HETE, 15-HEPE, 17-HDHA. Values are given as ng per 50 mg organ. LOD of the metabolites is indicated, if applicable. **Statistics**: Data are shown as mean ± SEM. The number of biological replicates is *n* = 5 for O-ISO and *n* = 5-6 for O-EX. Unpaired, two-tailed Student's *t*-tests with or without Welch-correction were performed for indicated comparisons.

Figure S5: Raw pictures of proteome profiler membranes and circulatory proteome changes.

a.

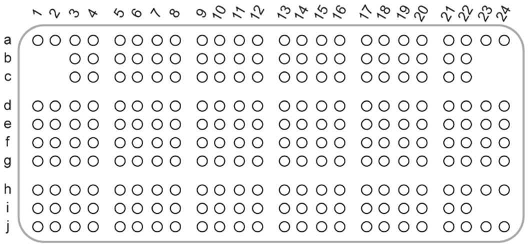

b.

spot	legend
A1-A2	reference spots
A3-A4	adiponectin
A5-A6	amphiregulin
A7-A8	angiopoietin-1
A9-A10	angiopoietin-2
A11-A12	angiopoietin-like 3
A13-A14	CD257
A15-A16	CD93
A17-A18	MCP-1
A19-A20	MIP-1α/β
A21-A22	RANTES
A23-A24	reference spots
B3-B4	MRP-1
B5-B6	eotaxin
B7-B8	MCP-5
B9-B10	TARC
B11-B12	MIP-3β
B13-B14	MIP-3α
B15-B16	SCYA21
B17-B18	MDC
B19-B20	CD14
B21-B22	CD40
C3-C4	CD160
C5-C6	chemerin
C7-C8	chitinase 3-like 1
C9-C10	CD142
C11-C12	C5a
C13-C14	complement factor D
C15-C16	CRP
C17-C18	fractalkine
C19-C20	KC
C21-C22	MIP-2
D1-D2	MIG
D3-D4	IP-10
D5-D6	I-TAC
D7-D8	BCA-1
D9-D10	CXCL16
D11-D12	cystatin C
D13-D14	DKK-1
D15-D16	CD26
D17-D18	EGF
D19-D20	endoglin
D21-D22	endostatin
D23-D24	fetuin A
E1-E2	FGF acidic
E3-E4	FGF-21
E5-E6	Flt-3 ligand
E7-E8	Gas 6
E9-E10	G-CSF
E11-E12	GDF-15
E13-E14	GM-CSF
E15-E16	HGF
E17-E18	ICAM-1
E19-E20	IFN-γ
E21-E22	IGFBP-1
E23-E24	IGFBP-2

spot	legend
F1-F2	IGFBP-3
F3-F4	IGFBP-5
F5-F6	IGFBP-6
F7-F8	IL-1α
F9-F10	IL-1β
F11-F12	IL-1ra
F13-F14	IL-2
F15-F16	IL-3
F17-F18	IL-4
F19-F20	IL-5
F21-F22	IL-6
F23-F24	IL-7
G1-G2	IL-10
G3-G4	IL-11
G5-G6	IL-12
G7-G8	IL-13
G9-G10	IL-15
G11-G12	IL-17A
G13-G14	IL-22
G15-G16	IL-23
G17-G18	IL-27
G19-G20	IL-28A/B
G21-G22	IL-33
G23-G24	LDL R
H1-H2	leptin
H3-H4	LIF
H5-H6	lipocalin-2
H7-H8	LIX
H9-H10	M-CSF
H11-H12	MMP-2
H13-H14	MMP-3
H15-H16	MMP-9
H17-H18	myeloperoxidase
H19-H20	osteopontin
H21-H22	osteoprotegerin
H23-H24	gliostatin
I1-I2	PDGF-BB
I3-I4	pentraxin 2
I5-I6	pentraxin 3
I7-I8	periostin
I9-I10	Pref-1
I11-I12	proliferin
I13-I14	PCSK9
I15-I16	RAGE
I17-I18	RBP4
I19-I20	Reg3G
I21-I22	resistin
J1-J2	reference spots
J3-J4	E-selectin
J5-J6	P-selectin
J7-J8	serpin E1
J9-J10	serpin F1
J11-J12	thrombopoietin
J13-J14	TIM-1
J15-J16	TNF-α
J17-J18	VCAM-1
J19-J20	VEGF
J21-J22	WISP-1
J23-J24	negative control

c.

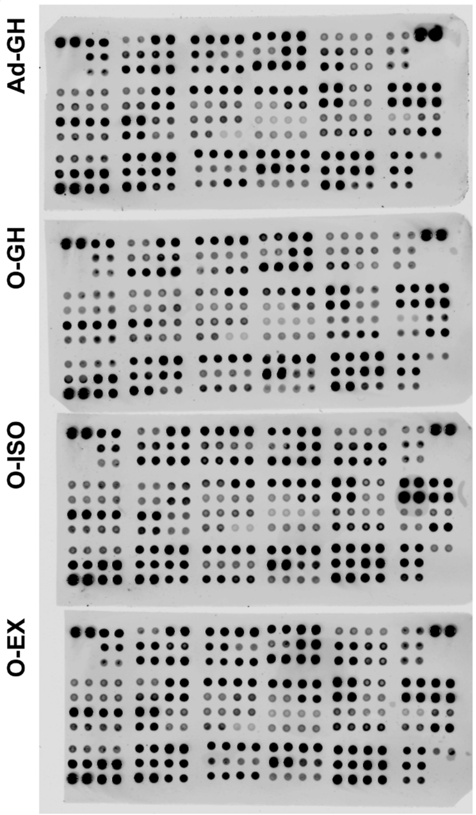

d.

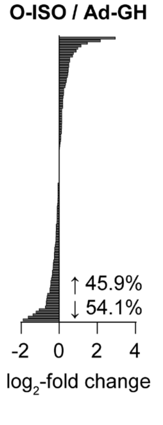

Fig. S5: Raw pictures of proteome profiler membranes and circulatory proteome changes. (a) Schematic layout of the proteome profiler overlay template used for the analysis of circulating proteins in pooled serum samples. **(b)** List of the 111 screened proteins allotted to their corresponding position on the membrane. **(c)** Pictures of the membranes used for the analysis of circulating serum proteins in pooled serum samples. **(d)** Log_2-fold changes of 111 screened, circulating proteins in pooled serum samples and percentages of up- or downregulated proteins for the comparison of aged, isolated mice (O-ISO) against adult, group-housed (Ad-GH) mice.

Figure S6: Representative chromatograms.

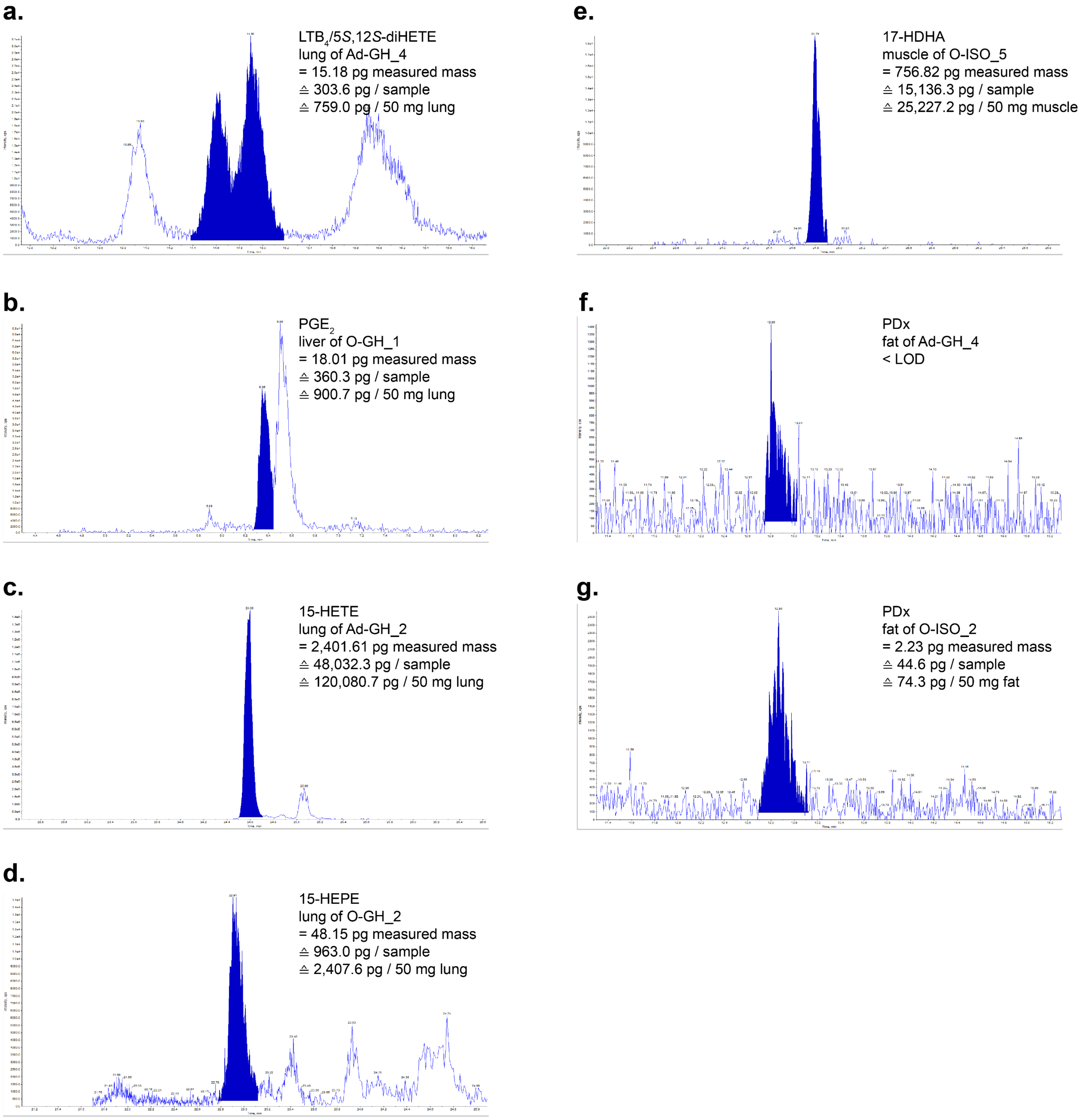

Fig. S6: Representative chromatograms. Representative, unsmoothed chromatograms including the integration of relevant metabolites **(a)** LTB_4/5*S*,12*S*-diHETE, **(b)** PGE_2, **(c)** 15-HETE, **(d)** 15-HEPE, **(e)** 17-HDHA, and **(f,g)** PDx. Given values represent the measured mass on column, the calculated mass in the sample, and the normalized amount per 50 mg organ.

Data S1: Screened PUFA metabolites, transitions, LOD and LLOQ. Comprehensive list of oxylipins that were analysed via UPLC-MS/MS including corresponding internal deuterated standard, specific Q1 and Q3 transitions that were used for quantification and respectively applied limits of detection (LOD) and lower limits of quantification (LLOQ) for each metabolite.

Data S2: Mass spectrometry (lipidomic) raw data. Searchable data table entailing the complete oxylipin raw data for each organ (brain, fat, heart, liver, lung, muscle, spleen, serum) from all experimental cohorts. Values below the limit of detection (LOD) or lower limit of quantification (LLOQ) were set to “not detectable (nd)” or “not quantifiable (nq)”, respectively.
